# Proteomic Changes Associated with Wooden Breast and White Striping in the *Pectoralis major* of Hubbard × Ross 708 Broilers: A Pilot Study

**DOI:** 10.3390/ani16111708

**Published:** 2026-06-03

**Authors:** Madeline C Karolak, Ahmet Yaman, Isain Zapata, Michael D Cressman, Macdonald P Wick

**Affiliations:** 1 Department of Animal Science, The Ohio State University, Columbus, OH 43210, USA; karolak.6@osu.edu (M.C.K.); ahmetyaman@ibu.edu.tr (A.Y.); cressman.2@osu.edu (M.D.C.); 2 Department of Poultry Science, Bolu Abant Izzet Baysal University, Bolu 14280, Türkiye; 3 Department of Biomedical Science, Rocky Vista University, Englewood, CO 80112, USA

**Keywords:** meat quality, poultry selection, proteomics

## Abstract

Wooden Breast (WB) and White Striping (WS) are prevalent myopathies affecting the *Pectoralis major* (PM) muscle of modern broiler chickens, with significant implications for animal welfare and meat quality. This pilot study aimed to characterize proteomic alterations associated with increasing severity of WB and WS in the PM of Hubbard × Ross 708 broilers. Muscle samples (*n* = 12) were previously categorized into four severity ranks based on established macroscopic criteria. Presently, proteins were extracted and analyzed using LC-MS/MS-based shotgun proteomics. Statistical comparisons between groups were performed using Generalized Linear Models. Fibronectin 1, fibrinogen alpha chain, musculoskeletal embryonic nuclear protein 1, glutathione S-transferase 2, calsequestrin-2, and endoplasmin emerged as potential biological markers and their prospective roles in the pathogenesis of these myopathies are discussed. Our findings suggest a progressive alteration of the muscle proteome with increasing disease severity, highlighting pathways related to muscle structure, metabolism, and stress response. These results aid in providing new insights into the molecular mechanisms underlying WB and WS and may inform future research on genetic and management strategies. As a pilot study, these findings warrant further investigation in larger cohorts and different commercial lines.

## Simple Summary

The broiler chicken industry is seeing more cases of White Striping (WS) and Wooden Breast (WB), which are unintended consequences of selection for increased pectoral muscle growth. These conditions lower breast meat quality through muscle damage and fibrosis, but the underlying causes and development are not fully understood. This research used LC-MS/MS-based proteomic analysis to observe changes in protein levels that are linked with increasing severity of an ischemia-related myopathy previously linked to WB in broiler pectoral muscle. This study found 24 proteins whose levels changed in relation to disease severity. Many of the identified proteins have not been previously associated with WS and WB and may represent new avenues of research to further characterize this myopathy. The findings support previously suggested ideas of myofiber damage and fiber-type changes, inflammation, oxidative stress, and calcium dysregulation. Overall, these results may contribute to ongoing efforts seeking to mitigate the impacts of WS and WB on poultry health and economics.

## 1. Introduction

The modern broiler industry has achieved remarkable gains in production efficiency and muscle yield through intensive selection for rapid growth, particularly of the *Pectoralis major* (PM) [1,2]. However, these genetic and management advances have been accompanied by a growing incidence of muscle disorders, including Spaghetti Meat (SM), White Striping (WS), and Wooden Breast (WB), which negatively impact animal welfare, meat quality, and economic returns [3,4,5,6,7,8]. SM is characterized by reduced structural integrity of the PM, resulting in the separation of the myofibrillar bundles [4,6,7]. Among these conditions, WB and WS are now recognized as the most prevalent myopathies affecting commercial broiler genetic lines, and their occurrence is associated with intensive selection in fast-growing genotypes raised under confinement.

Despite their significance, the underlying molecular mechanisms and pathogenesis of these myopathies remain incompletely understood. WS is macroscopically characterized by white, parallel striations of connective tissue and fat deposited along muscle fibers, while WB is distinguished by increased hardness, fibrosis, and pale discoloration in the PM [4,8,9,10]. Although both conditions often co-occur and share some pathological features, the distinction between WB and WS, as well as their potential overlap and relationship to commercial genetic lines and intensive production systems, remain subjects of ongoing debate [4,10]. Furthermore, the terminology surrounding “ischemia-related myopathy” often refers to the underlying condition associated with the manifestations of WS and WB. However, this term requires a clearer conceptual and methodological definition to advance the field. To our knowledge, no consensus definition exists for the use of ‘ischemia-related myopathy’ in the context of broiler muscle disorders.

Proteomic and transcriptomic analyses [11,12,13,14] of WS reported alterations in metabolic pathways, including carbohydrate metabolism, hypoxia, calcium homeostasis, inflammation, protein degradation, and fibrosis. Proteomic and transcriptomic analyses [15,16,17,18,19] of WB identified perturbations in carbohydrate metabolism, lipid metabolism, calcium homeostasis, muscle regeneration, oxidative stress, and fibrosis. However, most studies to date have relied on subjective scoring or limited biochemical markers, and few have systematically evaluated global proteomic changes across clearly defined severity grades.

Addressing these gaps, the present pilot study aims to characterize proteomic alterations associated with increasing severity of WB and WS in the PM of commercial Hubbard × Ross 708 broilers. By integrating objective severity grading with global LC-MS/MS-based proteomic analysis, we seek to explore molecular pathways and candidate protein markers that may inform both the classification and pathophysiology of these economically important myopathies. This pilot research does not intend to redefine WB, but rather to investigate protein abundance patterns associated with increasing severity of an ischemia-related myopathy in the tissue samples previously linked to WS and WB as described previously [4].

## 2. Materials and Methods

### 2.1. Animals and Sample Preparation

From a previous study [4], broiler chickens (Hubbard × Ross 708, mixed sex, *n* = 900) were raised under standard commercial conditions at the Ohio Agricultural Research and Development Center (OARDC, Wooster, OH, USA) Poultry Research Farm. Birds were wing banded and housed in floor pens. Birds had ad libitum access to a nutritionally complete diet, as well as unrestricted access to water through automatic drinker systems. During this previous trial [4], birds (3 per pen/*n* = 27) were randomly selected and slaughtered three times per week from 2 to 46 days of age. Randomization within pens was conducted to minimize selection bias. Slaughter procedures followed standard industry practices under The Ohio State University Institutional Animal Care and Use Committee (IACUC) approval. Carcasses were subsequently processed under consistent conditions prior to tissue collection.

Each PM sample was scored for myopathy severity on an increasing scale of 1 to 4, based on macroscopic features (hardness, visual white striations, pale color, etc.), following the classification system described by [4]. Briefly, 85 individuals displayed rank 1 as characterized by WS only, 116 individuals exhibited rank 2 as characterized by WS and surface hemorrhaging near the sternal apex, 95 individuals exhibited rank 3 as characterized by WS, surface hemorrhaging, and intramuscular hemorrhaging near the sternal apex, and 68 individuals displayed rank 4 as characterized by WS, surface hemorrhaging, intramuscular hemorrhaging, and ischemia near the periphery (Table 1).

The classical WB trait of tactile hardness was assessed in a subset of live birds to determine when WB-like abnormalities became detectable by palpation, but hardness scores were not used to define the severity ranks employed in the present study [4].

From the phenotyped population described above, three birds per severity rank (*n* = 12 total) were randomly selected for proteomic analysis. Selection was performed using a randomization procedure to ensure representative sampling within each severity category.

Whole muscle extracts were collected from the cranial region of the right *Pectoralis major* muscle to ensure sampling consistency across individuals. Approximately 100 to 200 mg of tissue was excised, trimmed of visible connective tissue and fat, and immediately transferred to a 2 mL microcentrifuge tube containing 1 mL of UTU extraction buffer, composed of 8 M urea, 2 M thiourea, 4% sodium dodecyl sulfate (SDS), 20 mM dithiothreitol (DTT), and bromophenol blue tracking dye (brilliant blue G-250). All reagents were prepared using analytical-grade chemicals Thermo Scientific, Waltham, MA, USA) and deionized water. The mixture was homogenized using a tissue homogenizer (OMNI International, Kennesaw, GA, USA) and the resulting homogenate was centrifuged at 17,000× *g* for 15 min at room temperature. The supernatant was collected and transferred to a separate 2 mL microcentrifuge tube and centrifuged again at 17,000× *g* for 10 min at room temperature; that supernatant was then transferred to a new 2 mL microcentrifuge tube. These supernatants were established as original stock solutions.

To prepare samples for downstream analysis, original stock solutions were diluted at a 1:3 ratio (1 part stock solution to 2 parts UTU buffer) to generate working stock solutions with reduced protein concentration and viscosity suitable for LC-MS/MS analysis. Original stock solutions were stored at −20 °C for long term preservation, while working stock solutions were maintained at 4 °C and used within a limited time window to minimize protein degradation.

### 2.2. Sequencing

Working stock solutions from three randomly selected samples per severity rank were submitted for liquid chromatography tandem mass spectrometry (LC-MS/MS)-based proteomic analysis at The Ohio State University Campus Chemical Instrument Center. Samples were prepared according to standardized protocols consistent with those described in previous studies [20] including reduction, alkylation, and enzymatic digestion of proteins into peptides prior to analysis.

Peptide separation was performed using a Nano-liquid chromatography-nanospray tandem mass spectrometry (capillary-LC/MS/MS) on a Thermo Scientific orbitrap Fusion mass spectrometer equipped with a nanospray FAIMS Pro™ Sources (Thermo Scientific, Waltham, MA, USA). The instrument was operated in positive ion mode, and electrospray ionization parameters were optimized to ensure stable spray and high sensitivity. Peptides were loaded onto a reversed-phase analytical column and separated using a linear gradient of increasing organic solvent concentration. Mass spectrometry data were acquired in data dependent acquisition mode, in which precursor ions detected in the full scan were sequentially selected for fragmentation and MS/MS analysis. Raw spectral data were processed using Proteome Discoverer (version 2.4.1.15, Thermo Scientific), with database searching conducted via Mascot Daemon (version 2.7.0, Matrix Science Ltd., London, United Kingdom) against the most recent UniProt *Gallus gallus* protein database. Search parameters included appropriate mass tolerances for precursor and fragment ions, as well as specified variable and fixed modifications consistent with sample preparation methods. A decoy database was also searched to determine the false discovery rate (FDR), and peptides were filtered at 1% FDR. Proteins identified with at least two unique peptides were considered as reliable identification.

### 2.3. Statistical Analysis

Normalized spectral count data derived from LC-MS/MS analysis of working stock samples were used as a semi quantitative measure of protein abundance. Data normalization procedures were applied to account for variation in sample loading and instrument performance across runs.

Protein level statistical analyses were conducted using generalized linear models, with myopathy severity rank treated as the independent variable. Each protein identified across samples was analyzed independently after aligning and matching protein identifiers across sequencing outputs. Model assumptions were evaluated prior to formal analysis, including assessment of residual distributions and homogeneity of variance using graphical methods such as residual plots and normal probability plots.

All statistical assessments were performed using SAS/STAT v.9.4 (SAS Institute Inc., Cary, NC, USA). Statistical significance was declared with a confidence threshold of 95%. Given the exploratory nature of the proteomic analysis and the relatively small sample size per group, no formal adjustment for multiple comparisons was applied. Therefore, reported *p* values should be interpreted with caution and considered as indicative rather than confirmatory evidence of differential protein abundance.

## 3. Results

LC-MS/MS-based proteomic analysis identified 24 proteins with significant (*p* < 0.05) abundance associations in relation to increasing severity rank of ischemia-related myopathy. Average normalized spectral counts by rank for the proteins are presented in Table 2. All identified proteins showed significant differences in abundances between rank 1 and rank 4 with an overarching increasing abundance trend in association with increasing ischemia-related myopathy severity.

**Figure 1:**
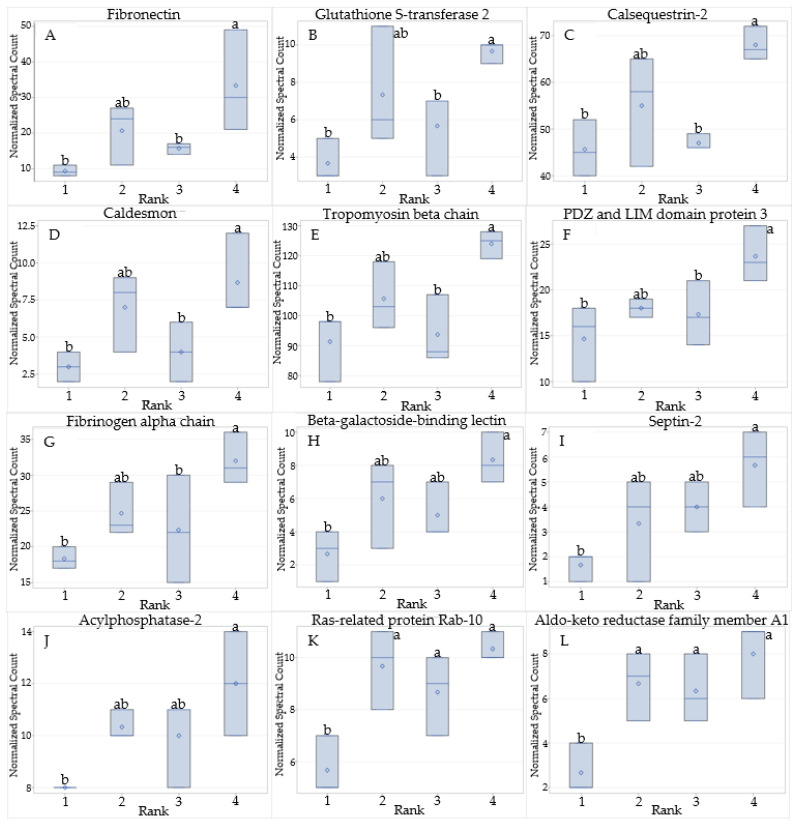
Distribution graphs displaying range of normalized spectral counts of proteins with significant differences (*p* < 0.05) found by LSMean comparison between rank 1 and rank 4 and rank 3 and rank 4. (**A**) Fibronectin. (**B**) Glutathione S-transferase 2. (**C**) Calsequestrin-2. (**D**) Caldesmon. (**E**) Tropomyosin beta chain. (**F**) PDZ and LIM domain protein 3. (**G**) Fibrinogen alpha chain. (**H**) Beta-galactoside-binding lectin. (**I**) Septin-2. (**J**) Acylphosphatase-2. (**K**) Ras-related protein Rab-10. (**L**) Aldo-keto reductase family 1 member A1. Pairwise comparisons assessments are presented using literals where ranks that do not share the same literal, are different at a threshold of *p* ≤ 0.05.

**Figure 2:**
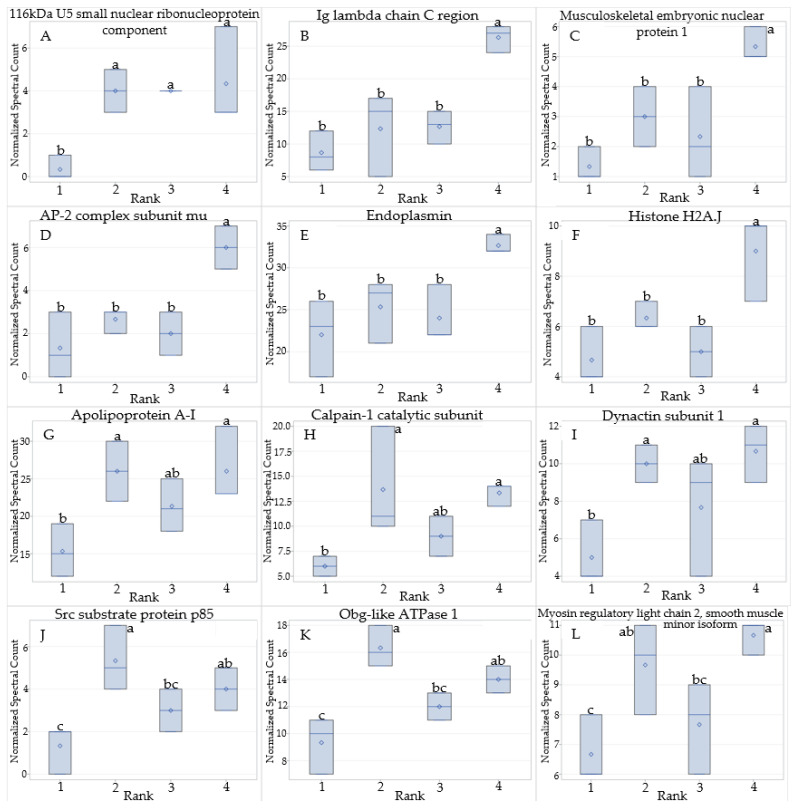
Distribution graphs displaying range of normalized spectral counts of proteins with significant differences (p<0.05) found by LSMean comparison between rank 1 and rank 4 and rank 3 and rank 4. (**A**) 116 kDa U5 small nuclear ribonucleoprotein component. (**B**) Ig lambda chain C region. (**C**) Musculoskeletal embryonic nuclear protein 1. (**D**) AP-2 complex subunit mu. (**E**) Endoplasmin. (**F**) Histone H2A J. (**G**) Apolipoprotein A-I. (**H**) Calpain-1 catalytic subunit. (**I**) Dynactin subunit 1. (**J**) Src substrate protein p85. (**K**) Obg-like ATPase 1. (**L**) Myosin regulatory light chain 2, smooth muscle minor isoform. Pairwise comparisons assessments are presented using literals where ranks that do not share the same literal, are different at a threshold of *p* ≤ 0.05.

Abundance trends across all ranks are presented in Figure 1 and Figure 2. Differences in abundances between adjacent ranks were more limited: nine proteins differed between rank 1 and rank 2, two proteins differed between rank 2 and rank 3, and 13 proteins differed between rank 3 and rank 4. Lastly, six proteins were found to have different abundances between rank 2 and rank 4, and three proteins were found to have different abundances between rank 1 and rank 3. Overall, LC-MS/MS-based proteomic analysis of objective ranks of ischemia-related myopathy in association with WB has uncovered relationships between protein abundance and increasing severity ranking but does not imply causality. Considering the limited sample size and the absence of formal multiple testing correction, this work should be regarded as exploratory and hypothesis-generating. The identified proteins represent candidates that require validation in independent, larger datasets.

## 4. Discussion

This pilot study suggests that increasing myopathy is associated with directional changes in muscle protein abundance. Rather than redefining WS or WB, these findings provide hypothesis-generating molecular/cellular associations linked to increasing ischemia-related myopathy severity within a phenotypic continuum previously described in the literature. Investigating protein abundance in fast-growing broilers may support a better mechanistic understanding of these myopathies and assist in mitigating their negative impacts on welfare and production. Previous research [4] proposed a phenotypic framework and ranking system describing the progression from WS to WB. This pilot study, by integrating the objective ranking system with global proteomic profiling, aids in extending this framework by providing preliminary insight into the pathophysiology of these myopathies. The use of this objective ranking system is crucial to the investigation of this myopathy’s development to better consolidate information observed across many studies. Many of the differentially expressed proteins identified herein have not been previously associated with WS and WB and may provide new avenues of research to further characterize this myopathy. Nevertheless, these findings may not directly generalize to other genotypes, management systems, or unaffected flocks because all samples were drawn from a WS/WB-affected commercial flock and no lesion-free controls were included. Moreover, though insignificant shifts between adjacent ranks may appear inconclusive, numerical differences still help to illustrate bigger picture trends that may be compatible with notions of metabolic shifts in these affected birds.

### 4.1. Muscle Regeneration and Fiber-Type Switching

WS and WB involve injury to the myofibers of the PM and attempts to recover. The selection for rapid PM growth in broilers results in muscle fiber hypertrophy that is greater than the surrounding connective tissue can accommodate, leading to reduced muscle capillary density, decreased muscle energy reserves, impaired energy metabolism, and hypoxia [7,21,22]. This damage invokes satellite cell-mediated repair mechanisms to regenerate myofibers to their original state [7,23,24,25,26]. Activation requires an appropriate niche environment and vascularization to the satellite cells [7,25,26,27]. In rapidly growing broiler chickens, however, the number of satellite cells available for regeneration and repair is depleted from self-donation to support myofiber hypertrophy [7,27]. Additionally, hypoxia and ischemia greatly limit satellite cell activation [7,28]. Two proteins integral to the contractile framework and regenerative process—tropomyosin beta chain (Tmβ) and musculoskeletal embryonic nuclear protein 1 (MUSTN1)—increased with myopathy severity. Tmβ is found in greater abundance in slow twitch chicken skeletal muscle as opposed to the alpha chain counterpart. MUSTN1 is a nuclear protein associated with embryonic development, postnatal growth, and skeletal muscle regeneration in avian species and has been shown to have increased expression in broilers when compared to layer counterparts [29], suggesting a role in skeletal muscle hypertrophy through skeletal muscle satellite cells. The increase in these proteins with increasing myopathy severity agrees with previous findings [30] and supports the possibility of fiber type switching in myopathic pectoral muscles due to the muscle no longer being able to regenerate to its previous state.

### 4.2. Inflammation

Inflammation is a key response to muscle damage in WS and WB. Fibronectin (FN1) is the ‘master organizer’ of the extracellular matrix and influences the balance between differentiation and regeneration of cells, increasing during states of morphogenesis, inflammation, repair, and fibrosis [24]. FN1 increased with increasing myopathy severity, which has also been observed when comparing unaffected birds to those affected by WS and WB [16,17,31]. This trend suggests increasing instances of tissue repair, inflammation, and fibrosis. Fibrinogen alpha chain (FGA), which is involved in blood clotting following injury and wound healing [32,33], also showed increased abundance with increasing severity, which agrees with the physiological manifestations of increased bruising as previously documented [4]. This trend contrasts with prior reports of downregulation of the coagulation pathway including FGA [34], and may be due to differences in scoring methodology, age at sampling, genetic background, and critically, the use of an objective ranking system in our study.

### 4.3. Oxidative Stress and Calcium Homeostasis

Oxidative stress is the consequence of an imbalance between the production of reactive oxygen species (ROS) and antioxidant neutralization, leading to cellular damage. Within skeletal muscle, ROS are produced from excessive muscle contraction, damaging cell structures such as the SR and altering proteins and membrane lipids [35,36,37]. Glutathione S-transferase 2 (GSTM2), an antioxidant expressed in chicken muscle tissue, detoxifies carcinogens and ROS, and regulates ryanodine receptor (RyR) Ca++ channels of skeletal and cardiac muscle in an activity-dependent manner [35,36,37]. GSTM2 increased with increasing myopathy severity. Upregulation of GSTM2 was also reported in broilers affected by WS and WB when compared to unaffected birds [38]. Considering the role of GSTM2 in both detoxification and Ca++ storage/release, the increase across rank may be consistent with attempts to mitigate the consequences of metabolic dysregulation and hypoxia, which can include increased reactive species and a stressed sarcoplasmic reticulum (SR) with impaired Ca++ homeostasis. Additionally, Calsequestrin-2 (CASQ2), a key Ca++ binding protein found within the lumen of the chicken junctional SR and involved in Ca++ storage within the SR [39], also increased with increasing myopathy severity. The increase in abundance across rank may be compatible with the notion of the tissue attempting to control the overabundance of Ca++ from a stressed SR. This trend contrasts with the previous literature which reported no difference in abundance between unaffected and WB affected birds [18]. Considering the tactile method reported [18] and the trend observed herein, the discrepancy in report may be accounted for if rank 3 and rank 4 birds were alternatively scored with the same severity.

In summary, 18 proteins were observed to have differential abundances related to rank of ischemia-related myopathy severity that have not previously been discussed in relation to WS, WB, or poultry in general. Taken together, and considering the limited sample size, absence of lesion-free controls, and lack of multiplicity correction, these rank-associated proteomic changes should be interpreted as hypothesis-generating. The identified proteins represent candidate markers and pathways that need to be validated in larger, independent datasets and, ultimately, linked to practical selection, nutrition, or management strategies. With these additional proteins now in observed association with this myopathy, new research avenues can be explored to better understand myopathy onset and progression.

### 4.4. Limitations and Future Directions

As a pilot study, a significant limitation is the lack of a true negative control, which constrains the generalizability of these findings. Photo documentation and sampling from the previous study [4] began at the time the first instances of WS were present on day 16 of age. Future research should document the entirety of the study to capture samples not yet affected by WS. Considering how early WS developed in the commercial broiler line, different populations could also serve as true negative controls, including Red Jungle Fowl, White Leghorns, or random bred control (RBC) genetic lines not selected for increased muscle growth to refine comparative analyses.

An additional limitation of the present study is the relatively small number of samples subjected to proteomic analysis, as only three birds per severity rank were sequenced. While this sampling approach is consistent with exploratory proteomic studies, it may limit the representativeness of each severity group and reduce the ability to fully capture biological variability within ranks. Future studies incorporating larger sample sizes per severity level would improve statistical power and enhance the robustness and generalizability of protein abundance patterns identified.

Additionally, this study only considered severity rankings for WS with respect to the presence of specific macroscopic traits such as bruising and ischemia and did not denote differential WB phenotypes through the classical methods of palpable hardness or histological analysis and therefore were not used to define severity groups. Including degree of WS severity by striping density in future studies would provide an even finer differentiation of protein abundance in association with myopathy severity. Considering the association-based nature of the proteomic approach, causality cannot be inferred. Despite these limitations, the use of an objective and reproducible severity ranking system based on macroscopic ischemic and hemorrhagic features and a global proteomic approach enabled the identification of coordinated protein abundance patterns across severity. The observation of stepwise protein abundance changes across severity ranks aids in strengthening the biological relevance of the associations, assisting in providing a novel molecular/cellular perspective on increasing ischemia-related myopathy severity previously linked to WB. Lastly, a complimentary transcriptomic study could be conducted to further elucidate relative RNA abundances with increasing myopathy severity. This subsequent study would highlight how gene expression is altered with myopathy progression and can be compared with the current proteomic information to uncover more about the transcription process in relation to ischemia-related myopathy.

## 5. Conclusions

This pilot study provides preliminary evidence that increasing severity of ischemia-related myopathy in broiler PM is associated with distinctive changes in the muscle proteome. These findings are compatible with previous work on myofiber damage, inflammation, and metabolic dysregulation, but should be viewed as exploratory. The identification of candidate proteins may inform future biomarker development and mechanistic studies, but further validation in larger and more diverse populations is essential before practical applications can be realized. Proteins such as FN1, FGA, MUSTN1, and GSTM2 emerge as candidate tissue markers of rank-associated changes and potential targets for future biomarker development that might be incorporated into more objective WS/WB ranking systems. The coordinated changes in GSTM2, CASQ2, and endoplasmin across ranks support the rational for developing nutritional (e.g., antioxidant, Ca-handling modulators) or genetic strategies that specifically target SR/Ca stress in WB-prone lines. Overall, these data also provide mechanistic insights, though dedicated applied studies are still needed to translate these findings into concrete interventions that reduce WS/WB prevalence or economic loss.

## Figures and Tables

**Table 1:** White Striping and Wooden Breast macroscopic severity grading system. From the total phenotypes population described previously [4], 3 birds per rank (*n* = 12 total) were randomly selected for proteomic analysis in the present study.

Rank (Increasing Severity)	WS Present	Surface Hemorrhage	Intramuscular Hemorrhage	Ischemia	Palpable Hardness Used to Define Rank?	*n* [4]
1	Yes	No	No	No	No	85
2	Yes	Yes (sternal apex)	No	No	No	116
3	Yes	Yes (sternal apex)	Yes (near sternal apex)	No	No	95
4	Yes	Yes	Yes	Yes (periphery)	No	68

**Table 2:** Average normalized spectral counts for proteins by severity rank. Pairwise comparisons assessments are presented using literals where ranks that do not share the same literal, are different at a threshold of *p* ≤ 0.05.

Protein	Average Normalized Spectral Count			
	Rank 1	Rank 2	Rank 3	Rank 4
Fibronectin	9.33 b	20.67 ab	15.67 b	33.33 a
Glutathione S-transferase 2	3.67 b	7.33 ab	5.67 b	9.67 a
Calsequestrin-2	45.67 b	55.00 ab	47.00 b	68.00 a
Caldesmon	3.00 b	7.00 ab	4.00 b	8.67 a
Tropomyosin beta chain	91.33 b	105.67 ab	93.67 b	124.00 a
PDZ and LIM domain protein 3	14.67 b	18.00 ab	17.33 b	23.67 a
Fibrinogen alpha chain	18.33 b	24.67 ab	22.33 b	32.00 a
Beta-galactoside-binding lectin	2.67 b	6.00 ab	5.00 ab	8.33 a
Septin-2	1.67 b	3.33 ab	4.00 ab	5.67 a
Acylphosphatase-2	8.00 b	10.33 ab	10.00 ab	12.00 a
Ras-related protein Rab-10	5.67 b	9.67 a	8.67 a	10.33 a
Aldo-keto reductase family 1 member A1	2.67 b	6.67 a	6.33 a	8.00 a
116 kDa U5 small nuclear ribonucleoprotein component	0.33 b	4.00 a	4.00 a	4.33 a
Ig lambda chain C region	8.67 b	12.33 b	12.67 b	26.33 a
Musculoskeletal embryonic nuclear protein 1	1.33 b	3.00 b	2.33 b	5.33 a
AP-2 complex subunit mu	1.33 b	2.67 b	2.00 b	6.00 a
Endoplasmin	22.00 b	25.33 b	24.00 b	32.67 a
Histone H2A.J	4.67 b	6.33 b	5.00 b	9.00 a
Apolipoprotein A-I	15.33 b	26.00 a	21.33 ab	26.00 a
Calpain-1 catalytic subunit	6.00 b	13.67 a	9.00 ab	13.33 a
Dynactin subunit 1	5.00 b	10.00 a	7.67 ab	10.67 a
Src substrate protein p85	1.33 c	5.33 a	3.00 bc	4.00 ab
Obg-like ATPase 1	9.33 c	16.33 a	12.00 bc	14.00 ab
Myosin regulatory light chain 2, smooth muscle minor isoform	6.67 c	9.67 ab	7.67 bc	10.67 a

## Data Availability

Curated datasets can be made available with a reasonable request to the corresponding author.
